# Biosynthesis of Vitamin C by Yeast Leads to Increased Stress Resistance

**DOI:** 10.1371/journal.pone.0001092

**Published:** 2007-10-31

**Authors:** Paola Branduardi, Tiziana Fossati, Michael Sauer, Roberto Pagani, Diethard Mattanovich, Danilo Porro

**Affiliations:** 1 Dipartimento di Biotecnologie e Bioscienze, Università degli Studi di Milano-Bicocca, Milano, Italy; 2 School of Bioengineering, University of Applied Sciences, Vienna, Austria; 3 Institute of Applied Microbiology, University of Natural Resources and Applied Life Sciences, Vienna, Austria; Baylor College of Medicine, United States of America

## Abstract

**Background:**

In industrial large scale bio-reactions micro-organisms are generally exposed to a variety of environmental stresses, which might be detrimental for growth and productivity. Reactive oxygen species (ROS) play a key role among the common stress factors–directly-through incomplete reduction of O_2_ during respiration, or indirectly-caused by other stressing factors. Vitamin C or L-ascorbic acid acts as a scavenger of ROS, thereby potentially protecting cells from harmful oxidative products. While most eukaryotes synthesize ascorbic acid, yeast cells produce erythro-ascorbic acid instead. The actual importance of this antioxidant substance for the yeast is still a subject of scientific debate.

**Methodology/Principal Findings:**

We set out to enable *Saccharomyces cerevisiae* cells to produce ascorbic acid intracellularly to protect the cells from detrimental effects of environmental stresses. We report for the first time the biosynthesis of L-ascorbic acid from D-glucose by metabolically engineered yeast cells. The amount of L-ascorbic acid produced leads to an improved robustness of the recombinant cells when they are subjected to stress conditions as often met during industrial fermentations. Not only resistance against oxidative agents as H_2_O_2_ is increased, but also the tolerance to low pH and weak organic acids at low pH is increased.

**Conclusions/Significance:**

This platform provides a new tool whose commercial applications may have a substantial impact on bio-industrial production of Vitamin C. Furthermore, we propose *S. cerevisiae* cells endogenously producing vitamin C as a cellular model to study the genesis/protection of ROS as well as genotoxicity.

## Introduction

Micro-organisms maintain optimal growth (and productivity) within a reasonably broad range of physiological conditions, due to a variety of responses that have evolved to cope with many types of environmental insult. Cellular defense mechanisms are able to avoid molecular damages in a wide range of environmental conditions. However, this balance can be disturbed severely in industrial scale bio-reactions where micro-organisms are generally exposed to a variety of environmental stresses [1], [2]. In addition to unfavorable exterior conditions the heavy metabolic burden imposed by an efficient production process is another cause for cellular stress. Regardless of their origin, stresses on micro-organisms can have various effects, including lower metabolic activity, growth rate, or productivity or decreased viability. In an industrial process, wherein the micro-organism is used as a means for production, the mentioned effects lead finally to a lower concentration of the product, lower productivity, or a decreased yield. Stress is therefore an undesirable phenomenon, and techniques for minimizing it-particularly in industrial processes-are highly desirable.

Reactive oxygen species (ROS) play a key role among the common stress factors [3]–[5].

This might be directly–by the generation of ROS due to the incomplete reduction of O_2_ during respiration-or indirectly–when ROS generation is caused by other stressing factors–metabolical or environmental. Most eukaryotic organisms produce l-ascorbic acid (L-AA or vitamin C), a powerful, water-soluble antioxidant as scavenger of ROS [6]–[8] to prevent or at least alleviate deleterious effects caused by ROS. However, yeast cells naturally lack the ability to produce L-AA. Instead, erythro-ascorbic acid, a structurally related compound with chemical properties very similar to those of L-AA, is the molecule occurring to a low extent in yeast cells [9]. Its role for stress resistance has been shown, but to which extend it is important is still a question for scientific debate [9]–[11].

Here, we report for the first time the biosynthesis of L-AA by metabolically engineered *S. cerevisiae* cells starting from D-glucose. We show that the endogenous biosynthesis of ascorbic acid in yeast and its scavenger role against ROS leads to an improved cell viability of the recombinant cells during growth under various stress conditions.

## Results

### Construction of recombinant S. cerevisiae strains able to convert D-glucose into L-ascorbic acid

**Figure 1:**
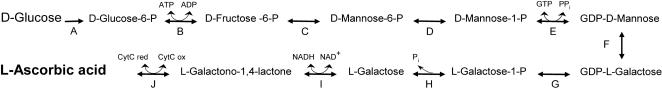
Ascorbic acid biosynthetic pathway. Schematic representation of the pathway of L-AA production from D-glucose in plants. The following enzymes are involved: A, hexokinase (2.7.1.1), B, glucose-6-phosphate isomerase (5.3.1.9), C, mannose-6-phosphate isomerase (5.3.1.8), D, phosphomannomutase (5.4.2.8), E, mannose-1-phosphate guanylyltransferase (2.7.7.22), F, GDP-mannose-3,5-epimerase (5.1.3.18), G, GDP-L-galactose phosphorylase (E.C.C not assigned), H, L-Galactose 1-phosphate phosphatase (3.1.3.25), I, L-galactose dehydrogenase, J, L-galactono-1,4-lactone dehydrogenase (1.3.2.3).

Animals and plants employ two different metabolic pathways to synthesize L-ascorbic acid [12], [13]. The plant pathway (shown in fig 1) was chosen for L-AA production in yeast cells for two reasons: First of all, GDP-mannose, a key intermediate in this pathway, is naturally produced in yeasts for cell wall construction [14]. Secondly, the last two steps for biosynthesis of L-AA in plants show similarity to the pathway of production of erythro-ascorbic acid in yeasts. Useful enzymes are therefore already present within the cells. It has been demonstrated that yeast strains incubated in the presence of L-galactose are able to produce l-ascorbic acid [15]. This capacity can be enhanced by overexpression of the endogenous *ALO1* gene and the heterologous *Arabidopsis thaliana LGDH* gene. In this case, L-AA is even accumulated extracellularly [16] upon incubation of the cells with L-galactose.

Only the three enzymatic activities converting GDP-D-mannose into L-galactose are therefore missing in yeast cells. To close this gap, the following genes were cloned and expressed under the control of the TPI promoter (Table 1): *AtME* (*A. thaliana* mannose epimerase), converting GDP-D-mannose into GDP-L-galactose and *AtMIP/VTC4* (*A. thaliana* myo-inositol phosphatase/L-galactose-1-P-phosphatase), converting L-galactose-1-P into L-galactose. Since the enzyme responsible for the conversion of GDP-L-galactose into L-galactose was only recently identified [17], alternative pyrophosphorylases converting substrates similar to GDP-L-galactose were sought for in the database. L-fucose guanylyl-transferase (*FGT)* from *Rattus norvegicus* [18] appeared suitable and was finally expressed. Additionally, *AtLGDH* (*A. thaliana* L-galactose dehydrogenase) which allows the conversion of L-galactose into L-galactono-1,4-lactone and *ScALO* (*S. cerevisiae* D-arabinono-1,4-lactone oxidase), converting L-galactono-1,4-lactone into L-AA were co-expressed, to make sure that no bottleneck is created in the end of the pathway.

**Table 1:** List of expression plasmids constructed and used in this study*

Expression vector	Promoter	Expressed protein	Plasmid status	Selection	Transformed strain
p012 MIP	*Sc* TPI	*At Myo-Inositol Phosphatase/L-Galactose-1P Phosphatase*	INT	*Sc URA3*	BY4742
p012*b*T ME-MIP	*Zb* TPI/*Sc*TPI	*At GDP-Mannose-3*′,*5*′*-Epimerase/ At Myo-Inositol Phosphatase/L-Galactose-1P Phosphatase*	INT	*Sc URA3*	GRF18U
p022 LGDH	*Sc* TPI	*At L-Galactose Dehydrogenase*	INT	*Sc HIS3*	BY4742/GRF18U
p042 ALO	*Sc* TPI	*Sc D-Arabinono-1,4-lactone oxydase*	INT	*Sc LEU2*	BY4742/GRF18U
p062 ME	*Sc* TPI	*At GDP-Mannose-3*′,*5*′*-Epimerase*	INT	*Sc LYS2*	BY4742/GRF18U
pZ_3_ ME	*Sc* TPI	*At GDP-Mannose-3*′,*5*′*-Epimerase*	CEN	Kan^R^	GRF18U
pZ_5_ FGT	*Sc* TPI	*Rn GDP-L-Fucose Pyrophosphorylase*	CEN	Nat^R^	BY4742/GRF18U

Abbreviations: Sc: *Saccharomyces cerevisiae*; At: *Arabidopsis thaliana*; Rn: *Rattus norvegicus*; Zb: *Zygosaccharomyces bailii*;

TPI: Triose Phosphate Isomerase

*URA3, HIS3, LEU2, LYS2*: gene markers conferring growth to auxotrophic yeast strains in the absence of uracil, histidine, leucine and lysine, respectively.

Nat^R^: cassette conferring resistance to nourseotricine.

Kan^R^: cassette conferring resistance to Geneticin.

CEN and INT: centromeric and integrative plasmids, respectively.

* a complete description of plasmids construction is given in Materials and Methods

**Figure 2:**
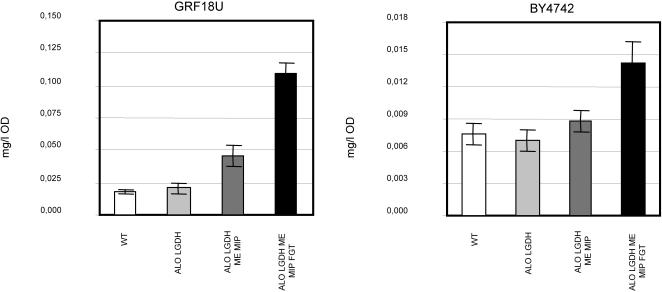
Conversion of D-Glucose into L-ascorbic acid (milligrams/liter/OD) by transformed *S. cerevisiae* GRF18U and BY4742 cells. All strains were grown on mineral medium (2% w/v glucose, 0.67% w/v YNB), starting with an initial OD660 of 0.05 for 18 h, when samples were taken and the concentration of L-ascorbic acid inside the cells was determined (GRF18U and BY4742 correspond to the parental strains transformed with the empty plasmids harboring in the productive strains the genes of the L-AA pathway). The control cells, as well as the cells expressing *ScALO1* and *AtLGDH* can not accumulate L-ascorbic acid starting from D-glucose, therefore measured values correspond to the endogenous erythro-ascorbic acid. The standard deviation bars correspond to the data obtained from independent clones, and from independent growth and antioxidant determinations. Please note the different scale of the ordinate axes in the two graphs.

The resulting integrative and/or centromeric expression plasmids (all five or, alternatively, all except the one harboring the *RnFGT* gene) were used to transform the *S. cerevisiae* strains GRF18U and BY4742 (see Table 1 and 2 for a complete list of plasmids and strains). Two different yeast strains were examined in parallel to elucidate the effect of a different genetic background. Independent clones of the obtained recombinant strains, together with their respective control strains, were grown in shake flasks with D-glucose as the sole carbon source. Their ability to convert D-glucose into L-ascorbic acid was measured by a chemical assay [19]. **Figure 2** clearly shows for both genetic backgrounds that the recombinant strains do produce low, but significant and reproducible amounts of vitamin C. Wild type cells as well as recombinant cells transformed with *ScALO* and *AtLGDH* were analyzed as negative control. For the cells expressing *ScALO* and *AtLGDH* we had previously proven a high accumulation of vitamin C, when the cells are incubated with L-galactose [16]. However, the basal level of endogenous antioxidants is similar for both the wild-type (first column) and these recombinant cells (second column) incubated with glucose. Adding two more of the genes from the L-AA metabolic pathway (*AtME* and *AtMIP)* the vitamin is already detectable (third column, for both graphs, strain BY4742[*ScALO AtLGDH AtME AtMIP*] and GRF18U[*ScALO AtLGDH AtME AtMIP*]). This result indicates the presence of endogenous pyrophosphorylase activities converting GDP-L-galactose into L-galactose-1-P. The level of L-ascorbic acid becomes even higher when the *RnFGT* gene is added (fourth column), proving the suitability of this enzyme for our purpose.

While the trend of ascorbic acid production for both strains is similar, the absolute values are significantly different, being almost one order of magnitude lower in the BY background (Fig. 2, left panel). Analogous, the contribution of the *RnFGT* gene is more effective in the GRF background (Fig. 2, right panel). On the other hand, while such difference could be related to the different genetic background of the two hosts, the basal level of antioxidants appears to be within the same order of magnitude in both yeast backgrounds. In no case vitamin C was detectable in the culture broth (data not shown).

### Effects of endogenous L-AA production on strain robustness: recombinant yeasts behavior under oxidative stress

**Figure 3:**
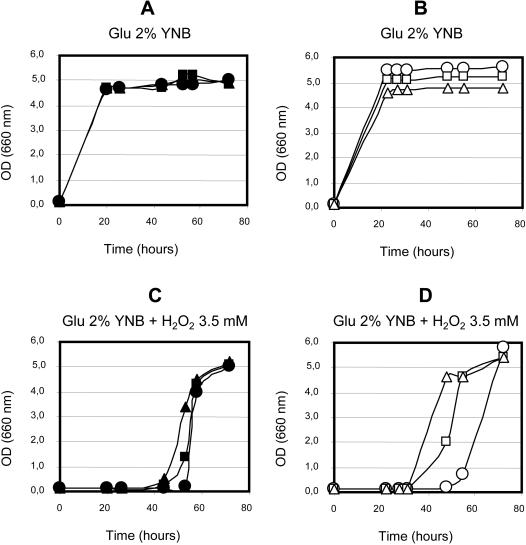
Growth curves of wild type and L-ascorbic acid producing yeasts under oxidative stress. Kinetics of growth of wild type and engineered strains GRF18U (left panels) and BY4742 (right panels) as inoculated in minimal glucose media without H_2_O_2_ (3A and 3B) or in presence of H_2_O_2_ 3.5 mM (3C and 3D). • GRF18U wild type; ▪ GRF18U[*ScALO AtLGDH AtME AtMIP*]; ▴ GRF18U[*ScALO AtLGDH AtME AtMIP RnFGT*]; ○ BY4742 wild type; □ BY4742[*ScALO AtLGDH AtME AtMIP*]; ▵ BY4742[*ScALO AtLGDH AtME AtMIP RnFGT*].

To test if the endogenous production of vitamin C could have beneficial biological effects, wild type BY4742, GRF18U and the corresponding recombinant L-AA producing strains were challenged by growing the cells under different stress conditions. Since vitamin C acts principally as an antioxidant, the first condition used to test the robustness of the engineered strains was oxidative stress. Yeast cells were inoculated at an initial optical density (660 nm) of 0.1 in the presence of different concentrations of H_2_O_2_ (ranging from 2 to 3.5 mM), and their growth was measured as optical density at specific intervals of time over about 80 hours, **Figure 3**. Cells were also inoculated in the same medium without H_2_O_2_, as a control. As expected, in the absence of H_2_O_2_ both wild type and recombinant strains grew well (Fig. 3a and 3b). When H_2_O_2_ was added to the medium, the growth of both GRF18U and BY4742 wild type strains was negatively affected proportionally to the hydrogen peroxide concentration. Low concentrations (2 mM) lead to a slight delay of growth (data not shown), while the highest H_2_O_2_ concentration (3.5 mM) lead to an almost complete growth inhibition (Fig. 3c and 3d). A similar behavior has been observed for yeast cells transformed with the Sc*ALO*1 and *AtLGDH* genes (data not shown). Significantly, under the same limiting conditions, all recombinant strains producing L-AA were able to resume growth about 35 hours after inoculation, showing a strong robustness and an increased tolerance to oxidative stress (Fig. 3c and 3d). Moreover, it becomes obvious that this aspect of strain robustness is directly correlated with the amount of L-AA produced (see Fig. 2); in fact, the strains engineered with all five genes necessary to complete the biosynthetic L-AA pathway exhibited even stronger tolerance (Fig. 3c and 3d, triangles) than the corresponding strains, lacking the FGT activity (Fig. 3c and 3d, squares). This phenomenon can be observed for both yeast genetic backgrounds, even if with a slight difference in timing, probably due to a different basal resistance of the two genetic backgrounds (phenomenon not further investigated). The BY background strain, in fact, seems to be more resistant, despite the generally lower ascorbic acid levels measured (see Fig. 2).

Similar results were obtained for cells growing in the presence of 2.5 and 3 mM H_2_O_2_. In these cases growth was resumed 20 and 25 hours after inoculation, respectively (data not shown), hence earlier and concentration dependent.

### Effects of endogenous L-AA production on strain robustness: recombinant strain behavior under acidic stress

To test if the improved strain robustness could be extended to other environmental constraints, wild type and engineered strains were subjected to acidic stress caused by inorganic or organic acids. These are typical environmental conditions occurring during either lab-batch and industrial fermentations.

**Figure 4:**
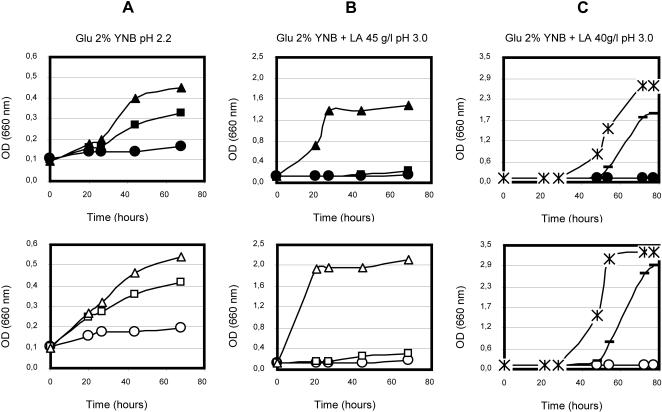
Growth curves of wild type and L-ascorbic acid producing yeasts under acidic stress. Growth curves of wild type and engineered GRF18U and BY strains (upper and lower panel, respectively) inoculated in minimal glucose media at low inorganic pH (panel A) or at low pH plus organic acid (lactic acid, 45g/l, panel B) or at low pH plus organic acid with L-AA added exogenously (panel C). Panels 4A and 4B: • GRF18U wild type; ▪ GRF18U[*ScALO AtLGDH AtME AtMIP*]; ▴ GRF18U[*ScALO AtLGDH AtME AtMIP RnFGT*]; ○ BY4742 wild type; □ BY4742 [*ScALO AtLGDH AtME AtMIP*]; ▵ BY4742 [*ScALO AtLGDH AtME AtMIP RnFGT*]; Panel 4C: • GRF18U wild type; ○ BY4742 wild type; − GRF18U or BY4742 wild type added with 30mg/l ascorbic acid; * GRF18U or BY4742 wild type added with 60 mg/l ascorbic acid.

To this purpose, we analyzed wild type and ascorbic acid producing strains grown in minimal medium brought to pH 2.2 with HCl addition (acidic stress induced by inorganic acid), or with lactic acid (45 g/l) at pH 3 (this value was chosen in order to have the organic acid almost completely in the undissociated form, since its pKa is 3.78). From the data presented in **Figure 4**, it is evident that also under these stress conditions the producing strains exhibit a strong robustness and increased tolerance when compared to control strain. At pH 2.2 (Fig. 4a) the growth rate of the wild type strains are significantly delayed in respect to the growth rate of the engineered strains.

Furthermore, the addition of 45 g/l of lactic acid to the medium has dramatic effects on the wild type strains, which are completely inhibited in growth, and also on the [*ScALO AtLGDH AtME AtMIP*] expressing cells which are severely compromised, while the recombinant strains expressing all genes for L-AA production are still able to grow (Fig. 4b). Interestingly, the effect of L-AA produced intracellularly is much more profound than the effect of vitamin C added to the culture broth. Fig. 4c shows the growth curve of wild type control cells in presence of lactic acid (40 g/l) at low pH. The addition of ascorbic acid relieves the growth inhibition evidently, but even with 60 mg/l the effect is not as profound as with the low amounts produced intracellularly. The growth is resumed much later even while less lactic acid was added to the culture.

### Correlation of endogenous L-AA concentration, strain robustness and viability

**Figure 5:**
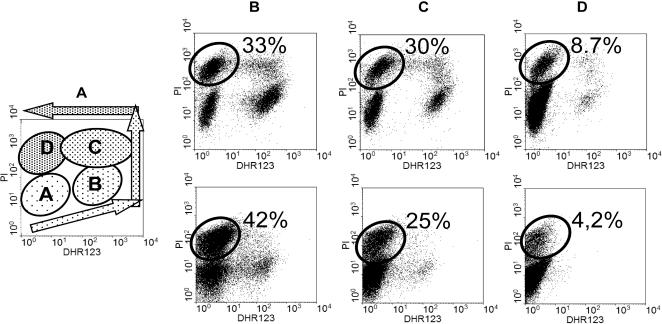
Flow cytometric analysis of wild type and vitamin C producing yeasts under oxidative stress. Panel 5A: schematic representation of the different subpopulations that can be observed in the following panels, where wild type (5B) and recombinant strains (5C and 5D) grown in minimal glucose medium added with H_2_O_2_ 3.0 mM were analyzed after DHR123 and PI staining (rodamine signal is reported in the abscissa and PI signal on the ordinate axes). Upper panels: GRF18U background. Lower panels: BY background. (B): wild type. (C): [*ScALO AtLGDH AtME AtMIP*] transformed cells. (D): [*ScALO AtLGDH AtME AtMIP RnFGT*] transformed cells

Having proven the positive effects on growth under stress conditions as described above, we wanted to deeper investigate the correlation between ascorbic acid endogenously produced by engineered yeast cells, enhanced tolerance to oxidative damage and cell robustness. The wild type and the recombinant GRF18U and BY strains were grown in minimal glucose medium with addition of 3.0 mM H_2_O_2_, following the same experimental protocol described in Figure 3. At the time in which all strains start to recover from the imposed stress (each with a different kinetic: approximately after 2–3 duplications from the preinoculum–as example see Figure 3), samples were taken. Each sample was stained with Dihydrorhodamine123 for the detection of ROS [20], and PI (propidium iodide) to detect severely damaged and/or dead cells [21]. Samples were analyzed with a flow cytometer. The resulting dot plots are compared in **Figure 5**. On the dot plots (DHR123 *vs.* PI) each individual cell is represented by a single dot and it is quite easy to recognize at least four distinct yeast subpopulations. A first healthy subpopulation (named A in Fig. 5a, used as a schematic representation for the benefit of the following ones), having only the background signal (autofluorescence) for both fluorochromes (low DHR and low PI signal); a second subpopulation (named B in Fig. 5a) of still viable but ROS accumulating cells (low PI, high rhodamine signal); a third subpopulation (named C in Fig. 5a) made of damaged cells displaying high ROS and high PI signals and finally a fourth subpopulation (named D in Fig 5a) of dead cells, presumably originated from subpopulation C by loosing all DHR signal. The data reported in the upper (GRF18U background) panels of Figure 5 (b, c, d) clearly show that in the wild type strain the oxidative stress induced by H_2_O_2_ strongly influences the intracellular ROS content and affects cell vitality. In fact, as clearly shown by Fig. 5b, a considerable portion of the analyzed cells (28%) shows high intracellular levels of ROS, and the fraction of dead cells (33%) exceeds that of viable cells (28%). By contrast, in the recombinant strains producing L-AA (Fig. 5c), we registered a clear reduction of ROS formation, (15% of the total population), together with a decrease of cell mortality (30%) and a consequent improved cell viability (44%). This trend is maximized in the strain expressing the *RnFGT* gene (Fig. 5d), where the majority of the cells (89.5%) is still viable and healthy, while ROS formation (1.1%) and cell mortality (8.7%) are almost negligible. As already observed for the growth curves in limiting conditions, also in this experiment there is a direct and positive correlation between the levels of vitamin C produced (see Fig. 2) and cell viability.

A corresponding result has been observed for the BY yeast background (lower panels). While the general trend for both strains is similar, in this case, accordingly to the higher robustness observed for this genetic background (compare the behaviors of the GRF18U and BY cells in Figure 3 and 4 ) a lower accumulation of ROS has been detected.

Taken together these data allow a more deeper analysis of the data shown in Figure 3 where increasing H_2_O_2_ concentration lead to longer delays of growth. These longer delays are clearly due to decreasing fractions of viable cells at the time of inolucum.

The same cells analysed by flow cytometry can also be analysed with a fluorescence microscope for ROS accumulation. In the wild type strain, cells with a strong and granular rhodamine staining are quite frequent and well represented, while in the producing strain almost no signal for ROS is detectable (data not shown).

All the described data demonstrate that the endogenous production of the antioxidant L-AA protects cells from oxidative damage, scavenging the intracellular production of ROS and/or increasing cell viability.

## Discussion

We report for the first time the biosynthesis of vitamin C by recombinant *S. cerevisiae* cells starting from D-glucose. Accumulation of ascorbic acid was proven to be successful in two different strains (Figure 2). Furthermore, we proved that the intracellular accumulation of L-AA leads to an improved robustness of the recombinant yeasts during growth under different stress conditions. The recombinant yeasts that are functionally transformed to produce L-ascorbic acid generate lower levels of ROS (Fig. 5) and exhibit improved growth and a higher viability under conditions of organic (Fig. 4b) and inorganic (Fig. 4a) acid stresses as well as oxidative stress (Fig. 3c and d). These are physiological conditions often encountered during industrial large scale fermentations. For each yeast background, the degree of robustness seems to be directly related to the amount of intracellularly produced vitamin C (Fig. 2, 3 and 4). In accordance with our data it has been shown before that the addition of ascorbic acid to the growth medium has beneficial effects for heterologous protein production in yeasts [22].

Furthermore, the respective yeast strains are a major step for the development of a *cell factory* for the production of vitamin C itself, one of the most important specialty chemicals manufactured in the world. This aim has been long sought for, but never reached up to now [23]–[25]. In this respect, it is noteworthy that we have previously shown that (*i*) yeast cells do have one or more transporters which allow the accumulation of intracellularly produced ascorbic acid in the culture medium (i.e., thus greatly facilitating the purification procedures) and (*ii*) that *S. cerevisiae* cells are quite tolerant to high concentrations of ascorbic acid (at least 40–50 g/L) [16]. Consequently, the industrial production of ascorbic acid by a one-step fermentation from D-glucose appears closer then ever.

One final general consideration can be made considering the role of ROS. It is well known that the production of ROS is common for many types of cancer cells and that vitamin C has a positive effect in reducing the incidence of stomach, lung and colorectal cancer [26], [27]. On the other hand vitamin C can under certain circumstances even increase the generation of ROS and could have detrimental effects at least under certain circumstances [28]–[30]. Hopefully, *S. cerevisiae* cells endogenously producing vitamin C could represent a well suited cellular model to study the genesis/protection of ROS and genotoxicity in higher eukaryotic organisms.

## Materials and Methods

### Yeast strains, transformation, media and cultivation

The *S. cerevisiae* parental strains used in this study were GRF18U [31] (MATα; *ura3*; *leu2-3,112*; *his3-11,15*; cir^+^), and BY4742 (MATa; *ura3Δ0*; *his3Δ1*; *leu2Δ0*; *lys2Δ0*; cir^+^), EuroScarf Accession No. Y10000-http://www.rz.uni-frankfurt.de/FB/fb16/mikro/euroscarf). Yeast transformations were performed according to the LiAc/PEG/ss-DNA protocol [32] and both strains were transformed with one or more of the constructs described below, in parallel with the corresponding empty plasmid(s). The presence of the heterologous genes was confirmed by PCR analysis. For each set of transformation at least three independent clones were initially tested, showing no meaningful differences among them. The resulting *S. cerevisiae* strains constructed in this study are listed in Table 2, with their respective genotypes. In GRF18U the plasmid integration is locus specific (the auxotrophies were generated by multiple point mutations of the target genes), while integration is randomly directed in cells of the BY background.

**Table 2:** List of yeast strains constructed and used in this study

Strain	Genotype	Source
GRF18U	*MATa his3-11 his3-15 leu2-3 leu2-112 ura3 [NRRL Y30320]*	[25]
GRF18Uc	*MATa his3-11 his3-15 leu2-3 leu2-112 ura3 [pYX012; pYX022; pYX042]*	This study
GRF18U ALO LGDH	*MATa his3-11 his3-15 leu2-3 leu2-112 ura3 [p022AtLGDH; p042ScALO]*	[9]
GRF18U ALO LGDH ME MIP	*MATa his3-11 his3-15 leu2-3 leu2-112 ura3 [p022AtLGDH; p042ScALO; pZ_3_AtME;* *p012AtMIP]*	This study
GRF18U ALO LGDH *b*TME MIP	*MATa his3-11 his3-15 leu2-3 leu2-112 ura3 [p022AtLGDH; p042ScALO; p012bTAtME AtMIP]*	This study
GRF18U ALO LGDH *b*TME MIP FGT	*MATa his3-11 his3-15 leu2-3 leu2-112 ura3 [p022AtLGDH; p042ScALO; p012bTAtME AtMIP; pZ_5_RnFGT]*	This study
BY4742	MATα; his3Δ1; leu2Δ0; lys2Δ0; ura3Δ0 (EuroScarf AN. Y10000)	[26]
BY4742c	MATα; his3Δ1; leu2Δ0; lys2Δ0; ura3Δ0 *[pYX012; pYX022; pYX042; pYX062]*	This study
BY4742 ALO LGDH	MATα; his3Δ1; leu2Δ0; lys2Δ0; ura3Δ0 *[pYX012; p022AtLGDH; p042 ScALO; pYX062]*	This study
BY4742 ALO LGDH ME	MATα; his3Δ1; leu2Δ0; lys2Δ0; ura3Δ0 *[pYX012; p022AtLGDH; p042ScALO; p062AtME]*	This study
BY4742 ALO LGDH ME MIP	MATα; his3Δ1; leu2Δ0; lys2Δ0; ura3Δ0 *[p012AtMIP; p022AtLGDH; p042ScALO; p062AtME]*	This study
BY4742 ALO LGDH ME MIP FGT	MATα; his3Δ1; leu2Δ0; lys2Δ0; ura3Δ0 *[p012AtMIP; p022AtLGDH; p042ScALO; p062AtME; pZ_5_RnFGT]*	This study

Yeast cultures were grown in minimal synthetic medium (0.67% w/v YNB Biolife without amino acids) with 2% w/v of D-glucose as carbon source. When required, supplements such as leucine, uracil, lysine and histidine were added to a final concentration of 50 mg/l, while the antibiotic nourseotricine sulphate (*cloNAT*, WERNER BioAgents, Germany) was added to a final concentration of 100 mg/l.

In the case of growth under oxidative or acidic stress, the desired stress condition was imposed by adding different concentrations of H_2_O_2_ (specifically 2, 2.5, 3 and 3.5 mM) or of lactic acid (45 g/l) directly to the medium or by lowering the medium pH to 2.2 with HCl 6N. In the case of lactic acid addition, the pH value was adjusted to 3 with NaOH 4N. Yeast cells were inoculated at an initial optical density of 0.1 (always 660 nm where not differently specified) and then optical density was measured at specific intervals of time over at least 55 hours from the inoculum. Each experiment was repeated at least three times.

All strains were grown in shake flasks at 30°C and 160 rpm and the ratio of flask volume/medium was of 5/1.

### Gene amplification and expression plasmid construction

All genes for the reconstruction of the L-AA pathway in yeast were PCR amplified on a GeneAmp PCR System 9700 (PE Appl. Biosystems, Inc.) using Pwo DNA Polymerase (Roche Diagnostics) and following the manufacturer's instructions. Template DNA for *AtLGDH*, *AtME*, and *AtMIP* was a cDNA library from *A. thaliana* (ATCC 77500) [33]; template DNA for Sc*ALO1* consisted of 50 ng of genomic DNA from *S. cerevisiae* GRF18U, extracted with a standard method (according to [34], slightly modified) *RnFGT* was PCR amplified from a kidney cDNA library (Origene, CR-1003). The following primer pairs were used: for *ALO1:* ALO1for TTT CAC CAT ATG TCT ACT ATC C and ALO1rev AAG GAT CCT AGT CGG ACA ACT C; for *LGDH*,: LGDHfor ATG ACG AAA ATA GAG CTT CGA GC and LGDHrev TTA GTT CTG ATG GAT TCC ACT TGG; for *ME*: MEfor GCG CCA TGG GAA CTA CCA ATG GAA CA and MErev GCG CTC GAG TCA CTC TTT TCC ATC A; for *MIP*: Mipfor ATC CAT GGC GGA CAA TGA TTC TC and MIPrev AAT CAT GCC CCT GTA AGC CGC; for *FGT* FGTfor TAG GAC ATG GAG ACT CTC CGG GAA and FGTrev CTC AAT TAA GAT TTC TCT AAA TCA GAT TGT TTT TTA TTT GA.

The PCR fragments were sub-cloned into the pSTBlue-1 vector using the Perfectly Blunt Cloning kit (Novagen) and checked by sequence analysis. The obtained sequences were the same as those reported in Genebank except for MIP in which two silent point substitutions (A271T and T685G) were detected. Finally, the coding sequences were *EcoR*I cut and sub-cloned into *S. cerevisiae* expression vectors of the YX series (R&D Systems, Inc.) or derivates (pYX012*b*T and pYX062, generated for this study, see below) and into the centromeric expression vectors p*Z*_3,_ pZ_3_ *b*T [35] and pZ_5_ (this study, see below). In detail, *ALO1* was sub-cloned into pYX042 (integrative, *LEU*2 auxotrophic marker, *Sc*TPI promoter); *LGDH* was sub-cloned into pYX022 (integrative, *HIS*3 auxotrophic marker, *Sc*TPI promoter), and ME and MIP into pYX062 (integrative, *LYS*2 auxotrophic marker, PCR amplified with oligonucleotides LYS2for TGC CAG CGG AAT TCC ACT TGC and LYS2rev AAT CTT TGT GAA GCT TCG CAA GTA TTC ATT from *S. cerevisiae* genome and substituted to the *URA3* auxotrophic marker in the plasmid pYX012 *Dra*III/*Not*I cut and blunt ended, *Sc*TPI promoter) and pYX012 (integrative, *URA*3 auxotrophic marker, *Sc*TPI promoter) respectively. *ME EcoR*I cut and blunt ended was also sub-cloned into pZ_3_ *b*T (centromeric, G418^R^ dominant marker, *Zb*TPI promoter) *Xba*I cut, blunt ended: the expression cassette *b*T promoter-*AtME*-polyA terminator was then PCR amplified with oligonucleotides btTPIfor ATC GTA TTG CTT CAA TTC TTC TTC TTT TGTA and polyAKpnrev GGG GTA CCC CAG CTG GAG CTA GAC AAA GAC which has a recognition sequence for *Kpn*I at the 5′ site; the cassette was then subcloned into pSTBlue-1 vector, and, after sequencing, *Kpn*I excised and sub-cloned into pYX012-*MIP Kpn*I cut, resulting in the pYX012 *b*TME-MIP expression vector. This construct allowed the integration of two heterologous genes into the yeast genome at the same time by the use of single marker of selection. Finally, *RnFGT EcoRI* cut was sub-cloned into pZ_5_. This is a centromeric vector derived from pBR1 (pYX022 in which the ARS-CEN fragment from Ycplac33 has been inserted [35]). pBR1 was cut *Kpn*I and blunt ended, and the nourseotricine cassette (Nat^R^) *Pvu*II/*Sac*I cut and blunt ended from pAG25 [36], has been inserted. All the restriction and modification enzymes used were from New England Biolabs (Hitchin, Herts, UK) or from Roche Diagnostics (Mannheim, Germany). Standard procedures [37] were employed for all cloning purposes.

All plasmids generated and utilized for the present study are described in Table 1.

### Determination of L-ascorbic acid

For intracellular L-ascorbic acid determinations yeast cells were inoculated at an initial optical density of 0.05 in minimal medium and grown for about 18 hours in order to reach a mid-exponential phase of growth. Cells were then harvested by centrifugation at 4000 rpm for 5 min at 4°C, washed once with ice-cold distilled water and then resuspended in about three times volume of ice-cold 10% (w/v) trichloroacetic acid, vortexed vigorously, and kept in ice for 20 minutes. The supernatant was then cleared from cell debris by centrifugation. Ascorbic acid was determined spectrophotometrically following a method adapted from that of Sullivan et Clarke [16], [19]. We previously confirmed the identity of ascorbic acid by HPLC using yeast cells functionally transformed to produce ascorbic acid from L-galactose [16].

### Flow cytometric analyses

Reactive oxygen species (ROS) were detected by Dihydrorhodamine 123 as described in [20]. Cells were incubated with Dihydrorhodamine 123 (DHR 123, Sigma Chemical Co., St. Louis, MO, USA) for 2 h, washed twice with PBS buffer and subsequently resuspended in propidium iodide solution 0.46 mM for the identification of dead or severely compromised cells. Samples were then analyzed using a Cell Lab Quanta™ SC flow cytometer (Beckman Coulter, Fullerton, CA, USA) equipped with a diode laser (excitation wavelength 488 nm, laser power 22 mW). The fluorescence emission was measured through a 525–550 nm band pass filter (FL1 parameter) for DHR signal and through a 670 nm long pass filter (FL3 parameter) for PI signal. The sample flow rate during analysis did not exceed 600–700 cells/s. A total of 20,000 cells was measured for each sample. Data analysis was performed afterwards with WinMDI 2.8 software, build#13 01-19-2000 (Purdue University, Cytometry Laboratories [http://facs.scripps.edu/software.html]).

### Fluorescence microscopy

Samples of the same yeast cultures treated and FACS analyzed for ROS content were also directly analysed using a Nikon Eclipse 90i fluorescence microscope (excitation, 488 nm) using standard FITC filters. Images were taken using the software Metamorph 2.2.
